# Hepatic Deficiency of Augmenter of Liver Regeneration Exacerbates Alcohol-Induced Liver Injury and Promotes Fibrosis in Mice

**DOI:** 10.1371/journal.pone.0147864

**Published:** 2016-01-25

**Authors:** Sudhir Kumar, Jiang Wang, Richa Rani, Chandrashekhar R. Gandhi

**Affiliations:** 1 Department of Surgery, University of Cincinnati, Cincinnati, Ohio, United States of America; 2 Cincinnati VA Medical Center, Cincinnati, Ohio, United States of America; 3 Department of Pathology and Laboratory Medicine, University of Cincinnati, Cincinnati, Ohio, United States of America; 4 Department of Pediatrics, Cincinnati Children’s Hospital Medical Center, Cincinnati, Ohio, United states of America; Texas A&M Health Science Center, UNITED STATES

## Abstract

Why only a subpopulation (about 15%) of humans develops liver cirrhosis due to alcohol is a critical as yet unanswered question. Liver-specific depletion of augmenter of liver regeneration (ALR) protein in mice causes robust steatosis and hepatocyte apoptosis by 2 weeks; these pathologies regress subsequently with return of ALR expression even at lower than control levels, but the mice develop modest steatohepatitis by 8 weeks. We aimed to investigate whether chronic alcohol ingestion promotes excessive hepatic fibrosis in these ALR-deficient mice. Liver-specific ALR-deficient and wild type (WT) female mice (8–10 weeks old) were placed on 4% alcohol-supplemented or isocaloric diet for 4 weeks. Liver sections were examined for histopathology, and parameters of steatosis and fibrosis were quantified. The mRNA expression of alcohol dehydrogenase-1, acetaldehyde dehydrogenase-1 and cytochrome P450-2E1 increased in WT mice but decreased in ALR-deficient mice upon alcohol ingestion. While alcohol induced steatosis and mild inflammation in WT mice, ALR-deficient mice showed minimal steatosis, strong hepatocellular injury and inflammation, prominent ductular proliferation, and robust fibrosis. Compared to the WT mice, alcohol feeding of ALR-deficient mice resulted in significantly greater increase in hepatic TNFα and TGFβ, and oxidative stress; there was also hepatic iron accumulation, robust lipid peroxidation and mitochondrial DNA damage. Importantly, similar to ALR-deficient mice, lower hepatic ALR levels in human alcoholic liver cirrhosis were associated with increased iron content, reduced expression of alcohol dehydrogenase and acetaldehyde dehydrogenase, and elevated fibrogenic markers. We conclude that ALR deficiency or anomaly can play a critical role in alcohol-induced hepatic fibrosis/cirrhosis, mechanisms of which may involve dysregulation of alcohol metabolism and iron homeostasis, mitochondrial damage and oxidative injury.

## Introduction

Alcoholic liver disease (ALD), the most common chronic liver disease, is the 10th leading cause of death in the United States. ALD is characterized by histopathological developments including steatosis, steatohepatitis, progressive fibrosis, cirrhosis, and hepatocellular carcinoma; genetic and environmental factors have been postulated to contribute to such disease progression [1,2]. Interestingly, whereas animals and a majority of humans develop reversible steatosis or steatohepatitis due to chronic alcohol ingestion, about 15% alcoholics progress to advanced liver disease (i.e., fibrosis and cirrhosis). Why this human subpopulation is predisposed to develop alcohol-induced hepatic fibrosis/cirrhosis is not clear [1,2].

Augmenter of liver regeneration (ALR) is a ubiquitous protein, originally identified and purified from weanling and regenerating rat livers [3]. ALR, produced as a ~22 kDa native protein, appears to be post-translationally modified to 3 forms (~36, 38 and 40 kDa) [4, 5]; specific functions of the 3 forms are not known [3]. Although a protein with molecular weight of ~15 kDa and strong homology to ALR was cloned from human fetal liver [6, 7], the mouse, rat and human ALR separates as ~22 kDa protein(s) under reducing condition by Western blot analysis; no 15 kDa protein is observed (S1 Fig) [3]. These findings indicate that the 22 kDa, and not 15 kDa, ALR is a physiological (unmodified) native protein in the liver. Expression of ALR in unmodified adult rat liver [4] suggested that it might have physiological functions. Indeed, ALR is shown to function as sulfhydryl oxidase [8], cytochrome c reductase [9, 10], and inducer of protein Fe/S maturation [11]. ALR is also reported to suppress hepatic NK cell cytotoxicity [12] and to stimulate cytokine synthesis in Kupffer cells [13]. Furthermore, ALR is a hepatocyte survival factor as indicated by mitochondrial dysfunction/damage and cell death upon inhibition of its synthesis [14].

Recently, we developed a liver-specific conditional knockout of ALR in mouse and found that ALR depletion causes robust steatosis and apoptosis of hepatocytes within 2 weeks post-birth; with subsequent increase in hepatic ALR levels, steatosis regresses but the liver develops ductular proliferation, inflammation and evidence of fibrosis by 4 weeks, followed by reduced ductular proliferation, minimal steatosis, and moderate inflammation and fibrosis by 8 weeks [15]. Hepatic ALR levels are still much lower at 8 weeks [15]. Since hepatic ALR levels are also lower than normal in advanced ALD [15], we hypothesized that ALR deficiency or anomaly may determine the course of ALD accelerating from steatohepatitis to excessive fibrosis. Indeed, the results show that while alcohol ingestion causes steatosis in wild type (WT) mice, ALR-deficient mice develop only minimal steatosis but demonstrate impaired iron homeostasis and excessive fibrosis.

## Materials and Methods

Unless indicated otherwise, all chemicals were purchased from Sigma-Aldrich, St. Louis, MO.

### Ethanol feeding

This study was carried out in strict accordance with the recommendations in the Guide for the Care and Use of Laboratory Animals of the National Institutes of Health. The protocols were approved by the Committee of Ethics of the University of Cincinnati (Institutional Animal Care and Use Committee) (Protocol number: 12-10-16-01). All surgeries were performed under isoflurane anesthesia, and all efforts were made to minimize suffering. ALR-deficient mice (Alb-Cre;ALR^*floxed/floxed*^) and WT mice (Alb-Cre^-/-^;ALR^*floxed/floxed*^) on a B6.SV129 background [15] were bred at the University of Cincinnati Animal Facility. 8–10 week-old female mice were placed on Lieber-DeCarli ‘82 Shake supplemented with Pour liquid diet (Bio-Serv, NJ- Product F1259SP). After 2 days, 50% of the mice continued on this diet (pair-fed); the other 50% were placed on an equivalent diet (Product F1697SP) supplemented with 1.78% (v/v) (day 3 and 4), 3.38% (day 5 and 6) and 4% ethanol from day 7 (ethanol-fed). The diet consumption and body weight were recorded on alternate days, when the diet was renewed. At the end of 4 weeks of 4% ethanol feeding, mice were anesthetized with isoflurane, blood was drawn, and the liver excised, weighed and portions fixed in 10% buffered formalin, 2% paraformaldehyde or snap-frozen in liquid nitrogen.

### Histopathological evaluation of Liver

Formalin-fixed and paraffin-embedded liver sections were stained with hematoxylin/eosin (H/E) for histopathological evaluation by one of the authors (JW) who was blinded to the samples. Liver pathology was scored as described by Brunt [16].

### Biochemical and molecular analyses

Serum alanine aminotransferase (ALT) was measured using Infinity ALT reagent (Thermo DMA, Louisville, CO).

Hepatic lipids were extracted in chloroform/methanol (2:1; v/v). Phases were separated by adding 0.73% NaCl solution to attain chloroform/methanol/NaCl ratio of 1.3:1:0.92 (v/v). The chloroform (organic) phase was aspirated, evaporated under nitrogen, and dissolved in 5% Triton X-100. Triglycerides and cholesterol were quantified using assay kits from Stanbio Laboratories, Boerne, TX.

Lipid peroxidation was measured as described [15]. Briefly, the tissue was homogenized in 20 mM phosphate (pH 7.4) containing 5 mM butylated hydroxytoluene, mixed at 1:2 ratio with a solution containing 15% trichloroacetic acid, 0.37% thiobarbituric acid, 0.2 M HCl and 0.03% butylated hydroxytoluene, and boiled for 15 min. Thiobarbituric acid reactive substances were measured at 535 nm.

Hepatic glutathione content was quantified using an assay kit from Cayman Chemical Co, Ann Arbor, MI. Briefly, 1g liver tissue was homogenized in 5 ml ice-cold buffer consisting of 0.2 M 2-(N-morpholino)-ethanesulphonic acid, 0.05 M phosphate, and 1 mM EDTA, pH 6–7. Freshly deproteinized supernatants (50 μl), without or with 2-vinylpyridine, were mixed with 150 μl of freshly prepared assay cocktail to measure GSH and glutathione disulfide (GSSG), respectively. Absorbance was measured at 405 nm at 5 min intervals for 30 min.

TNFα, IL6 and IL10 levels were measured in liver lysates using mouse Luminex^®^ beadset kit (Millipore, Billerica, MA) (catalog# MCYTOMAG-70K-15**)** in Bio-plex 100 equipped with operating system 6.1 apparatus.

Hydroxyproline concentration was measured as described [15,17]. Briefly, 100 mg liver tissue was homogenized in 250 μl of 6M HCl. After incubation at 120°C for 24h, the homogenate was centrifuged (10,000g/5 min) at room temperature. 50 μl of the supernatant or standards (0–1000 μg/ml 4-hydroxy-L-proline) were incubated with 450 μl of chloramine-T solution (0.056M;1.27 g of chloramine-T dissolved in 20 ml 50% n-propanol and adjusted to 100 ml with acetate-citrate buffer) for 25 min at room temperature, then mixed with 500 μl of freshly prepared Ehrlich's reagent [1M p-dimethylaminobenzaldehyde in n-propanol/perchloric acid (2:1; v/v)]. After incubation at 65°C for 20 min to develop chromophore, absorbance was recorded at 550 nm in a Synergy^™^ H4 Multi-Mode Reader (Bio-tek^®^ Instruments, Inc., Winooski, VT).

Caspase-3 activity was measured using a fluorimetry-based assay kit (Sigma-Aldrich). The tissue was homogenized in cold lysis buffer (50 mM HEPES, pH 7.4, containing 150 mM NaCl, 20 mM EDTA, 0.2% Triton X-100, containing protease inhibitor cocktail) and incubated on ice for 30 min. The lysate was centrifuged (13000g/15 min/4°C), and the enzyme activity in the supernatant was measured by caspase-3-mediated release of the fluorescent 7-amino-4-methylcoumarin from the substrate acetyl-Asp-Glu-Val-Asp-7-amido-4-methylcoumarin (Ac-DEVD-AMC).

Carnitine palmitoyl transferase (CPT) activity was determined by measuring CoA-SH released from palmitoyl-CoA by thiol reagent 5, 5’-dithiobis-(2-nitrobenzoic acid) (DTNB) [15, 18]. Briefly, the tissue was homogenized in a solution containing 0.25M sucrose, 1mM EDTA, 0.1% ethanol, 0.1 mM sodium orthovanadate, 0.2 mM phenylmethylsulfonyl fluoride and 1% protease inhibitors mixture (Santa Cruz Biotechnology) (1:5; w/v) on ice, and centrifuged at 300g for 10 min at 4°C. The supernatant was further centrifuged at 12,000g for 5 min at 4°C. 10 μl of the supernatant was mixed with an equal volume of Tris-HCl-DTNB buffer (116 mM Tris-HCl, pH 8.0, containing 2.5 mM EDTA, 2 mM DTNB, and 0.2% Triton X- 100) and incubated for 5 min at 30°C in a 96-well plate. 10 μl of 1 mM palmitoyl-CoA was added, and the reaction was initiated by adding 2 μl L-carnitine solution (1.2 mM in 1 M Tris-HCl, pH 8.0). Optical density was recorded at 412 nm.

### Oil red-O and Sirius red Staining

Paraformaldehyde-preserved liver sections were allowed to dry on glass slides, then fixed in ice-cold 10% neutral buffered formalin. The sections were treated successively with absolute propylene glycol, pre-warmed oil red O and 85% propylene glycol, then rinsed in water, treated with Mayer’s hematoxylin, rinsed in water and cover-slipped with gelvatol. Deparaffinized liver sections were also stained with 0.1% sirius red (Direct Red 80) in saturated aqueous picric acid, and counterstained with H/E. Morphometric analysis and quantification were performed using ImageJ software (NIH).

### Immunostaining

Formalin-fixed liver sections (5 μm) were deparaffinized with xylene and rehydrated through series of graded alcohol then washed with 1.5% H2O2 in PBS for 20 min at 25°C to block endogenous peroxidases. The sections were treated with RNase (5 mg/ml in 50 mM Tris-HCl (pH 8.0), 10 mM EDTA) for 1h, blocked for 10 min at 25°C with 5% normal donkey serum in PBS containing 0.1% Triton X-100 and 3% BSA, then incubated overnight at 4°C with mouse monoclonal anti-8-oxoguanine Ab (1:400) (Abcam, Cambridge, MA), followed by HRP-conjugated rabbit anti-mouse IgG (secondary Ab). 8-oxoguanine was visualized using 3,3’-diaminobezidine substrate kit (Pierce Biotechnology, Inc., Rockford, IL). For alpha-smooth muscle actin (αSMA) or desmin, deparaffinized liver sections were treated with anti-αSMA or anti-desmin Ab (Abcam, Cambridge, MA), HRP-conjugated goat anti rabbit IgG secondary antibody, followed by 3,3’-diaminobezidine.

### RNA and Protein analysis

RNA was prepared from the frozen liver tissue and converted to cDNA using a reverse transcription system (Promega BioSystems, Sunnyvale, CA). Various mRNA transcripts were quantified by qRT-PCR using SYBR-Green in a 7300 real time PCR analyzer (Applied Biosystems, Grand Island, NY). Primer sequences are listed in Table 1.

**Table 1:** Quantitative Polymerase Chain Reaction Primer Sequences.

**Mouse**	**Forward (5’-3’)**	**Reverse (5’-3’)**
ACACA	AGCCAGAAGGGACAGTAGAA	CTCAGCCAAGCGGATGTAAA
ADH1	GCCGAAGCGATCTGCTAAT	AGGTGCTGGTGCTGATAAAG
ALDH1a1	GCAGCAGGACTCTTCACTAAA	CACTGGGCTGACAACATCATA
ALR	AATTGGGTCGCCACACCTG	GGCCAGCGTATGGAGGAAA
βactin	AGAGGGAAATCGTGCGTGAC	CAATAGTGATGACCTGGCCGT
Collagen 1	CCAGAGTGGAACAGCGATTAC	GATGCAGGTTTCACCAGTAGAG
CPT1α	ACTCTTGGAAGAAGTTCA	AGTATCTTTGACAGCTGGGAC
CYP2E1	GAAGTCTCTGGTTGACCCTAAG	AGGTCTCATGAACGAGGAATG
FAS	CTGCGGAAACTTCAGGAAATG	GGTTCGGAATGCTATCCAGG
GLRX5	GGGAGTCACTTCTGATTCTTGT	TTCTCCTCTGTCCATCATTTCC
IL6	CCGGAGAGGAGACTTCACAG	TCCACGATTTCCCAGAGAAC
IL10	CCCTGGGTGAGAAGCTGAAG	CACTGCCTTGCTCTTATTTTCACA
SREBP1c	AGCCCTCCACCAGGTAATAA	GGGTTCCCAGTCTACTCACTAA
TGFβ	GGTGGTATACTGAGACACCTT	CCCAAGGAAAGGTAGGTGATA
TNFα	CCCAGGTATATGGGCTCATACC	GCCGATTTGCTATCTCATACCAGG
**Human**	**Forward (5’-3’)**	**Reverse (5’-3’)**
ADH1	GTGCTCTACCTGTGCTGTGTTTG	CACCTCCATCAGTCATTTCCTTTA
ALDH1a1	TGTTAGCTGATGCCGACTTG	TTCTTAGCCCGCTCAACACT
βactin	TCACCCACACTGTGCCCATCTACG	CAGCGGAACCGCTCATTGCCAATG
Collagen I	TGGAGAGAGAGGTGAACAAGG	CATCACCCTTAGCACCATCG
TGFβ	CCCAGCATCTGCAAAGCTC	GTCAATGTACAGCTGCCGCA
TNFα	GGAGAAGGGTGACCGACTCA	CTGCCCAGACTCGGCAA

Liver protein lysates were prepared in RIPA lysis buffer containing protease inhibitors cocktail (Santa Cruz Biotechnology, Dallas, TX). Proteins were separated by SDS-PAGE and transferred to Immobilon^®^ PVDF membrane (Millipore, Billerica, MA). After immunoblotting with primary Abs [ALR/GFER (Proteintech Group, Inc, Chicago, IL), alcohol dehydrogenases-1 (ADH1), aldehyde dehydrogenases-1 (ALDH1), rabbit polyclonal anti-Bax or anti-Bcl2 (Cell Signaling Technology, Inc., Beverly, MA) washing and incubation with appropriate HRP-conjugated secondary Abs, protein bands were detected using ECL reagent (Thermo Fisher Scientific, Grand Island, NY). β-actin expression was assessed to ensure equal protein loading.

### Perls’ Prussian blue staining

Deparaffinized liver sections were washed in water, and incubated in pre-warmed 1:1 solution of 5% HCl and 5% potassium ferrocyanide for 5 min. The sections were rinsed in water, and counterstained with nuclear-fast red for 5 min, dehydrated, cleared and coverslipped.

### Acetaldehyde determination

Hepatic acetaldehyde was measured using Amplite^™^ Fluorimetric Aldehyde Quantitation kit (10052) (AAT Bioquest, Inc., Sunnyvale, CA). Briefly, the liver was homogenized in PBS, and centrifuged at 2,500 rpm for 10 min. 50 μl of the supernatant or 0–1 mM aldehyde (standards) was mixed with 50 μl of AldeLight^™^ Blue reaction mixture. Following incubation in solid black 96 well plate for 30 min, 25 μl of reaction buffer was added, and fluorescence was monitored at Ex/Em = 365/435 nm.

### Non-heme iron Content

Total hepatic non-heme iron content was measured in a microplate with bathophenanthroline-based procedure [19, 20]. Briefly, 100 mg of the tissue was digested at 65°C for 24h in 500 μl 0.3% trichloroacetic acid in 8 M HCl, then centrifuged at 10,000g for 5 min, and the supernatant (50 μl) was incubated for 5 min at room temperature with 150 μl of freshly prepared color reagent (a mixture of 15.6 g of sodium acetate in 45 ml water and 17 mg bathophenanthroline disulfonic acid and 22 mg L-ascorbic acid in 5 ml water). Absorbance was measured at 539 nm against reagent blank (bathophenanthroline chromogen) in a Synergy H4 Multi-Mode Reader (Bio-tek^®^ Instruments, Inc., Winooski, VT), and the concentration of iron was estimated from the absorbance for serially diluted standard iron solution (TraceCERT^®^ Fluka, Sigma-Aldrich, St. Louis, MO).

### Insulin determination

Serum insulin levels were quantified using an ultrasensitive mouse insulin ELISA kit (Crystal Chem, Downers Grove, IL). Briefly, 5 μl of serum samples or serially diluted insulin standards and 95 μl of sample diluent were placed in the antibody-coated wells of a microplate. After 2h at 4°C, the wells were washed five times with 200 μl of wash buffer, and 100 μl of horseradish peroxidase (HRP)-conjugated anti-insulin antibody was added per well. Following 30 min at room temperature, the wells were washed, and 100 μl of enzyme substrate solution was added. The reaction was allowed for 40 min in dark at room temperature to detect the HRP-conjugate, and stopped by adding 100 μl of stop solution (1N sulfuric acid) per well. The absorbance was measured at 450 nm (630 nm reference wavelength) in the Synergy^™^ 4 Multi-Mode Microplate Reader.

### Statistical analysis

The data were analyzed with Sigma Plot 12.0 (Systat Software, Inc., San Jose, CA). Statistical significance was determined using One Way ANOVA followed by Bonferroni’s multiple comparison tests. Kruskal–Wallis tests were conducted if the data did not have a normal distribution. Mann-Whitney U rank sum test was used to compare the pathological scores. A p value of <0.05 was considered as statistically significant.

## Results

### Body and liver weights of ethanol-fed ALR-deficient mice

**Fig 1:**
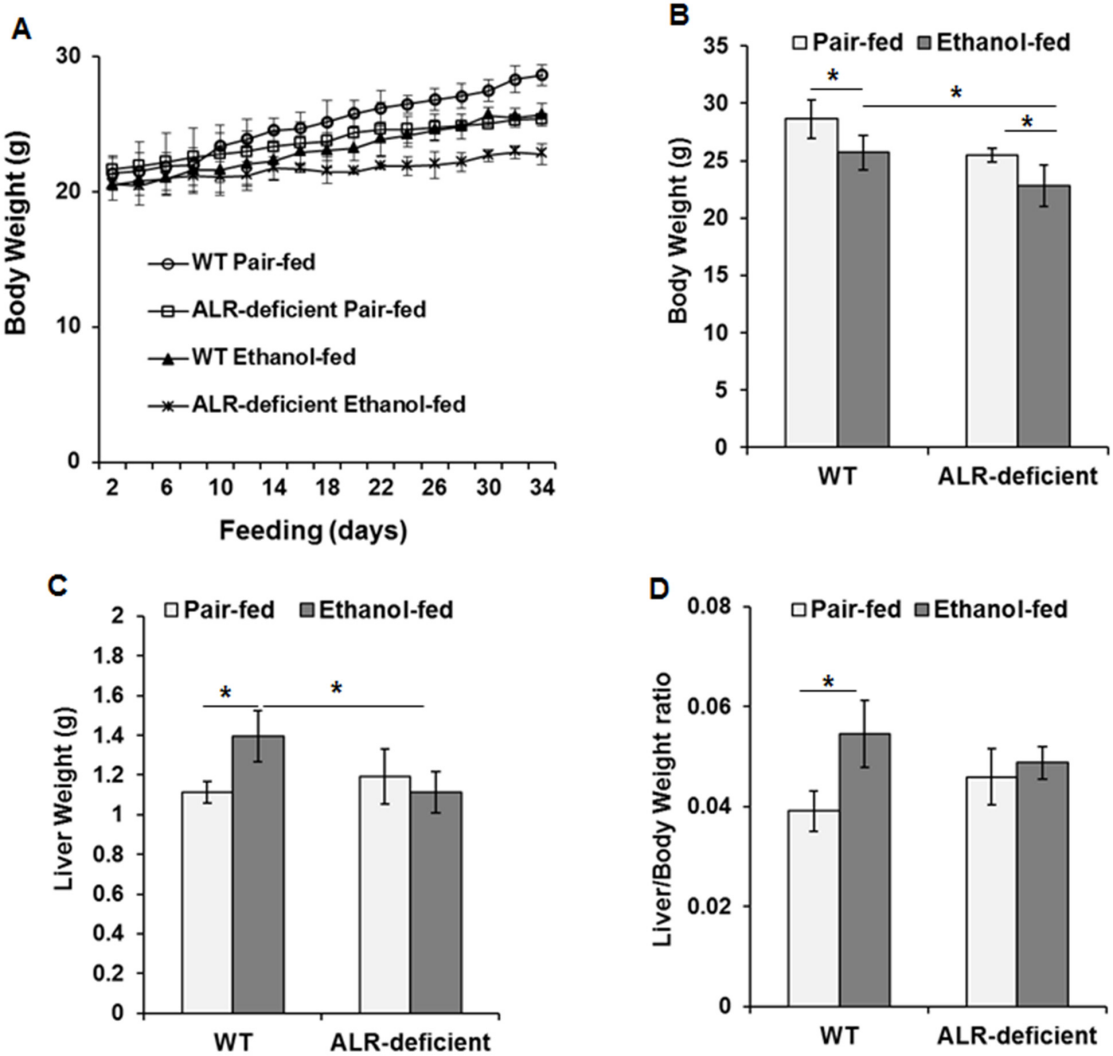
Effect of ethanol feeding on liver and body weight of WT and ALR-deficient mice. WT and ALR-deficient mice were placed on isocaloric- or ethanol-diet for 4 weeks. The mice were weighed on alternate days. Changes in **(A, B)** body weight, **(C)** liver weight and **(D)** liver/body weight ratio are shown. *p<0.05.

The weight gain of both ethanol-fed WT and ALR-deficient mice was lower than the pair-fed mice, magnitude of which being significantly greater for ALR-deficient mice (Fig 1A and 1B). The liver weight increased in ethanol-fed relative to pair-fed WT mice, whereas it remained unaltered in ALR-deficient mice (Fig 1C) resulting in significantly higher liver/body weight ratio in ethanol-fed than pair-fed WT mice but not ALR-deficient mice (Fig 1D).

### Alcohol reduces hepatic ALR in both WT and ALR-deficient mice

**Fig 2:**
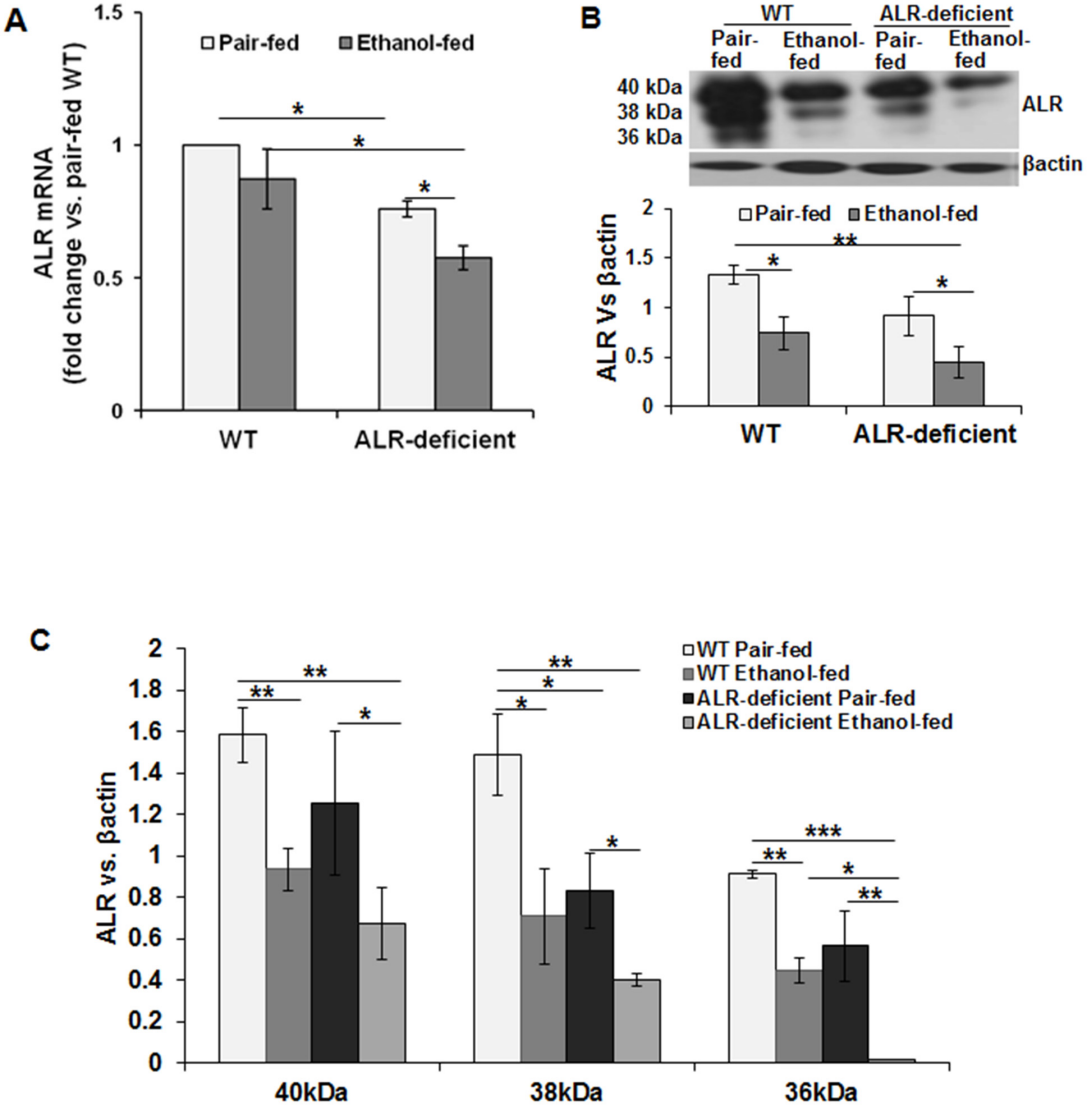
Effect of ethanol feeding on hepatic ALR in WT and ALR-deficient mice. WT and ALR-deficient mice were placed on isocaloric- or ethanol-diet for 4 weeks. ALR mRNA was quantified via qRT-PCR **(A)**. **(B, C)** Western blot analysis was performed to identify changes in individual ALR isoforms. The values shown are average ± SD of the ratio of individual ALR isoform vs. β-actin. *p<0.05; **p<0.005; ***p<0.0005.

Hepatic ALR mRNA and protein expression were lower in ALR-deficient than in WT mice (Fig 2). Whereas the hepatic ALR mRNA expression tended to decrease in ethanol-fed WT mice, the protein levels decreased significantly by about 50%. Hepatic ALR content was already 40% lower in pair-fed ALR-deficient mice than in WT mice; it decreased further at the end of ethanol feeding to about 30% of the pair-fed WT level. The decrease occurred in all 3 forms of ALR in both phenotypes upon ethanol feeding (Fig 2B and 2C).

### Ethanol-induced liver injury and steatosis in ALR-deficient mice

**Fig 3:**
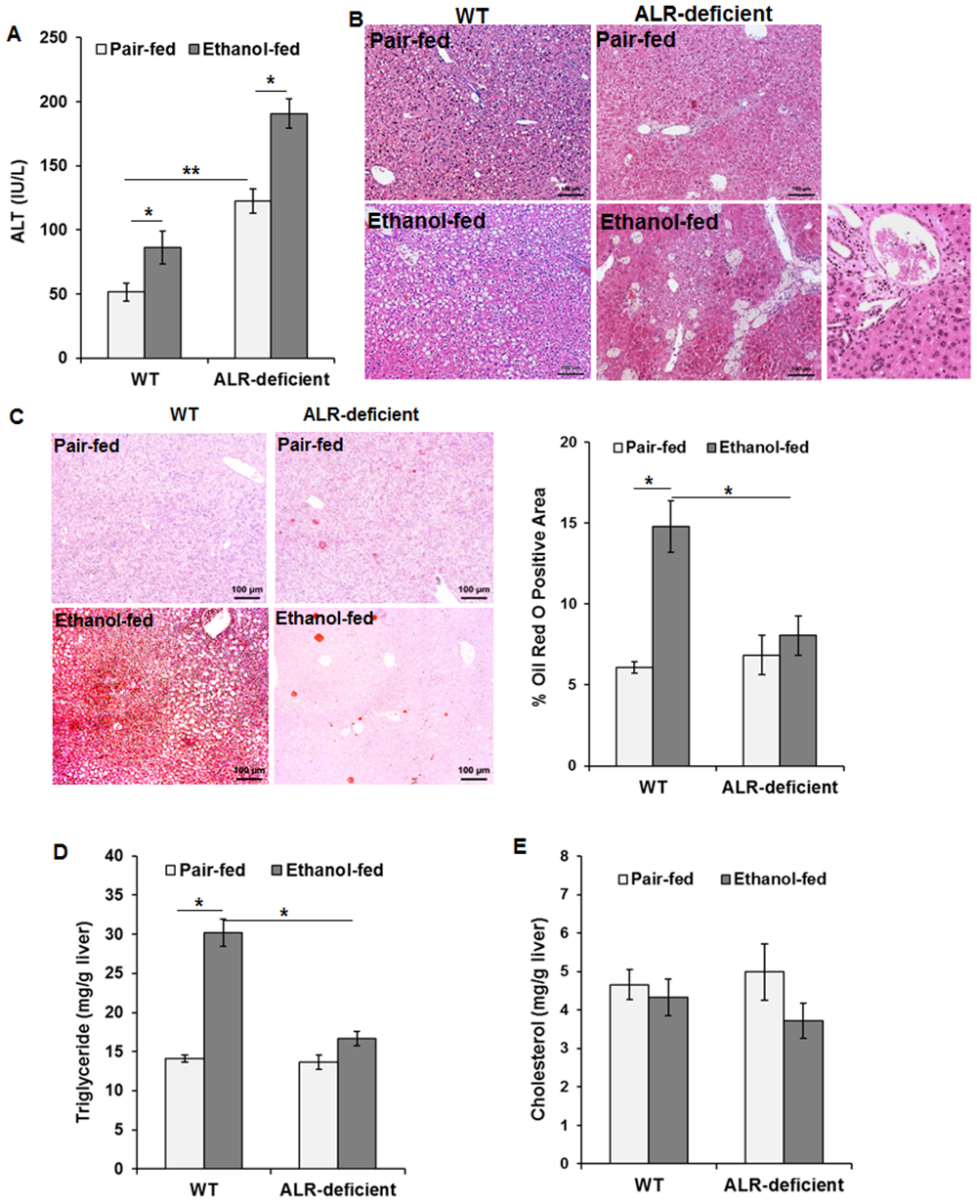
Effect of ethanol feeding on liver injury and steatosis in WT and ALR-deficient mice. WT and ALR-deficient mice were placed on isocaloric- or ethanol-diet for 4 weeks. **(A)** Serum ALT levels (average ± SD). *p<0.05; **p<0.005. **(B)** Representative H/E-stained liver sections show histopathological changes, including steatosis, inflammation, hepatocyte swelling, ballooning degeneration, lipogranulomas and ductular proliferation. Original magnification, x10. **(C)** Oil Red O staining and morphometric analyses depict marked steatosis in ethanol-fed WT mice but not in ALR-deficient mice. Hepatic triglyceride **(D)** and cholesterol **(E)** levels in ethanol-fed WT and ALR-deficient mice. The values shown are average ± SD. *p<0.05.

Compared to WT mice, serum ALT was higher in pair-fed ALR-deficient mice indicating underlying liver injury (Fig 3A). Ethanol feeding caused further increase in ALT in ALR-deficient mice. Ethanol-induced increase in ALT was relatively modest for WT mice (Fig 3A). Ethanol feeding caused very low level of inflammation in WT mice (Fig 3B). The modest inflammation observed in pair-fed ALR-deficient mice increased further upon ethanol feeding, especially in the portal region, accompanied by strong increases in liver cell injury (cell swelling, ballooning), pigmented macrophages, and lipogranulomas (Fig 3B; S2 Fig; Table 2). Interestingly, the mild ductular proliferation seen in ALR-deficient mice [15] before the start of treatment regressed in pair-fed mice but increased in alcohol-fed mice (Fig 3B; S2 Fig; Table 2). In chow diet-fed ALR-deficient mice also ductular proliferation was not apparent or minimal at 12 weeks (S3 Fig). Such ductular reaction has been documented in ALD, especially when fibrosis and cirrhosis develop [21].

**Table 2:** Histology scores in different groups of mice models.

Histological features	Histology score/Grade and Description	Histology Score/Grade±SEM^ƚ^			
		WT mice		ALR-deficient mice	
		Pair-fed	Ethanol-fed	Pair-fed	Ethanol-fed
**Steatosis**					
Microvesicular steatosis	0:none; 1:up to 33%; 2:33 to 66%; 3:>66%	0.00±0.00	5.00±2.80	1.66±1.66	5.00±3.50
Macrovesicular steatosis	0:none; 1:up to 33%; 2:33 to 66%; 3:>66%	5.50±2.60	25.0±8.50^a^	1.66±1.66	2.50±1.40^c^
Total steatosis	0:none; 1:up to 33%; 2:33 to 66%; 3:>66%	5.50±2.60	27.5±7.50^a^	3.33±3.33	7.50±4.30^c^
**Hepatocellular Injury**					
Swelling	0:none to minimal, 1:mild, 2:moderate, 3:severe	1.25±0.47	2.25±0.47	1.66±0.66^b^	2.75±0.25^c^
Ballooning degeneration	0:none, 1:few, 2:many, 3:extensive	0.00±0.00	0.75±0.75	0.66±0.66^b^	1.00± 0.40^c^
Pigmented macrophages	0:none, 1:few, 2:many, 3:extensive	0.00±0.00	0.00±0.00^a^	0.33±0.33^b^	2.25±0.47^c^
**Inflammation**					
Microgranulomas	0:absent, 1:occasional, 2:many, 3:extensive	0.00±0.00	0.00±0.00	0.00±0.00	0.00±0.00
Lipogranulomas	0:absent, 1:occasional, 2:many, 3:extensive	0.00±0.00	0.00±0.00^a^	0.00±0.00^b^	1.50±0.50^c^
Lobular inflammation	0:no inflammatory foci, 1:<2 inflammatory foci, 2:2–4 inflammatory foci, 3:>4 inflammatory foci	0.00±0.00	0.50±0.28^a^	1.33±0.33	1.00±0.00^c^
Portal inflammation	0:none to minimal, 1:mild, 2:moderate, 3:marked	0.50±0.28	0.50±0.28^a^	1.00±1.00^b^	2.00±0.00^c^
**Fibrosis**					
Stage	0:none, 1:mild, zone 3 or 3 & 2, 2:moderate, Stage 1 changes + portal/periportal, 3:bridging, 4:cirrhosis	0.00±0.00	0.25±0.25^a^	1.33±1.33^b^	3.00±0.40^c^
Pericellular fibrosis	0:none, 1:mild, zone 3 or 3 & 2, 2:moderate, Stage 1 changes + portal/periportal, 3:bridging, 4:cirrhosis	0.00±0.00	0.25±0.25^a^	1.00±1.00^b^	2.25±0.75^c^
**Other finding**					
Ductular proliferation^ǂ^		Absent	Absent	Minimal	Prominent

^**ƚ**^Data are presented as mean ± SEM (n = 4 for pair-fed and n = 6 for ethanol-fed mice). Statistical significance was calculated as follows: Macrovesicular and total steatosis (a Vs c, *p*<0.005); hepatocyte swelling (b Vs c, *p*<0.05); hepatocytes ballooning degeneration (b Vs c, *p*<0.05); pigmented macrophages (a Vs c, *p*<0.0005; b Vs c, *p*<0.0005); lipogranulomas (a Vs c, *p*<0.0005; b Vs c, *p*<0.0005); lobular inflammation (a Vs c, *p*<0.05); portal inflammation (a Vs c, *p*<0.0005; b Vs c, *p*<0.05); fibrotic stage and pericellular fibrosis (a Vs c, *p*<0.0005; b Vs c, *p*<0.005);

^ǂ^Histology scores were not calculated.

Ethanol-fed WT mice developed marked micro- and macrovesicular steatosis (Fig 3B; Table 2). In contrast, ALR-deficient mice failed to develop significant steatosis upon alcohol ingestion. These observations were congruent with strong oil red O staining (Fig 3C) and increase in hepatic triglycerides (Fig 3D) in WT mice, but not in ALR-deficient mice. Cholesterol levels were unaltered by ethanol feeding in both phenotypes (Fig 3E).

**Fig 4:**
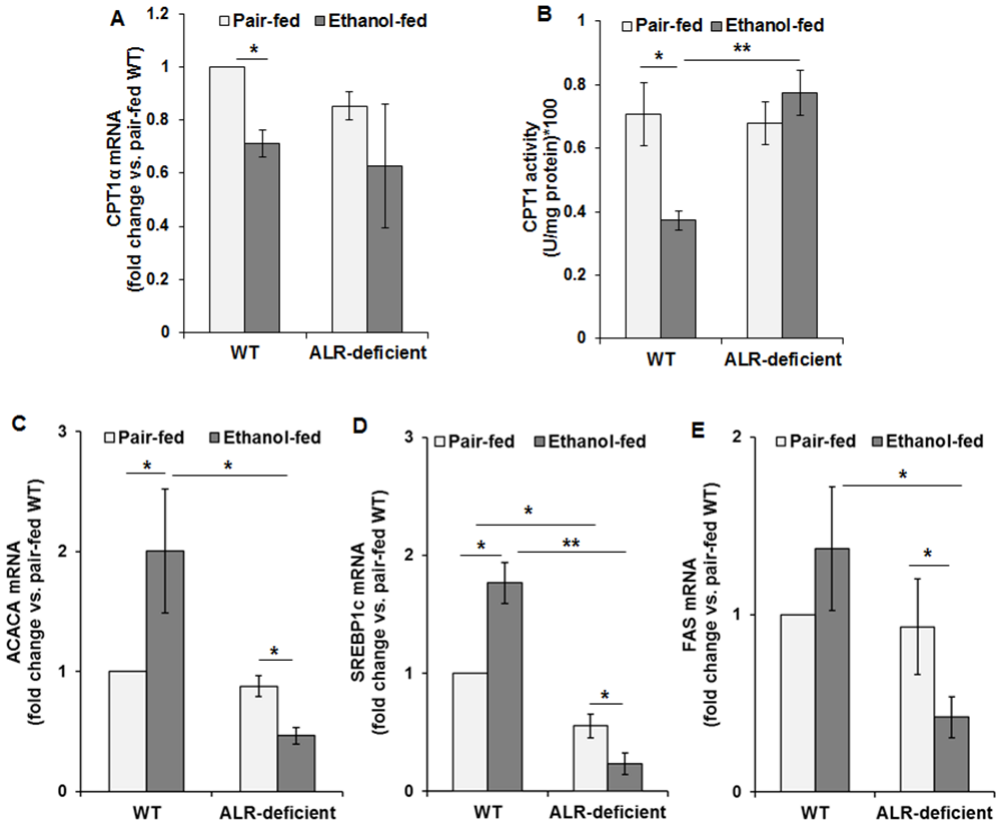
Effect of ethanol feeding on lipid-metabolizing enzymes in ALR-deficient mice. WT and ALR-deficient mice were placed on isocaloric- or ethanol-diet for 4 weeks. Hepatic **(A)** CPT1α mRNA and **(B)** CPT1 enzymatic activity, **(C)** ACACA mRNA, **(D)** SREBP1c mRNA, and **(E)** FAS mRNA expressions normalized with that of β-actin are shown. Average ± SD. *p<0.05, **p<0.005.

Compared to the pair-fed WT mice, hepatic mRNA expression as well as enzymatic activity of CPT1α (a protein that transports free fatty acids into mitochondria) decreased upon ethanol feeding (Fig 4A and 4B). CPT1α mRNA expression and activity in pair-fed ALR-deficient mice were similar to that in pair-fed WT mice, and did not change by alcohol feeding (Fig 4A and 4B).

The mRNA expression of acetyl-CoA carboxylase (ACACA), sterol regulatory element-binding protein 1c (SREBP1c) and fatty acid synthase (FAS) all increased in ethanol-fed relative to pair-fed WT mice (Fig 4C–4E). In contrast, expression level of these transcripts decreased significantly in ethanol-fed ALR-deficient mice.

### ALR deficiency and ethanol-metabolizing enzymes

**Fig 5:**
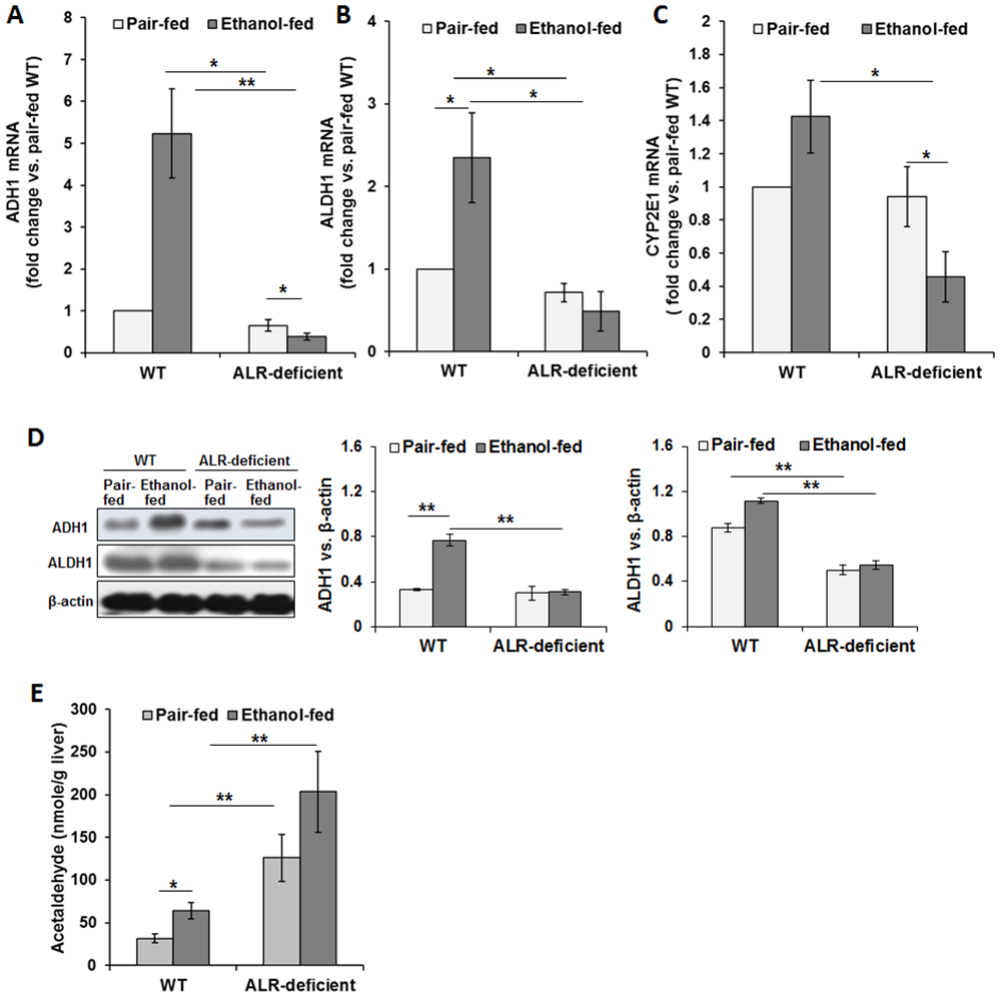
Ethanol-induced changes in the gene expression of alcohol-metabolizing enzymes in WT and ALR-deficient mice. WT and ALR-deficient mice were placed on isocaloric- or ethanol-diet for 4 weeks. Bar graphs show hepatic mRNA expression of **(A)** ADH1, **(B)** ALDH1 and **(C)** CYP2E1, normalized with β-actin. **(D)** Immunoblot shows protein levels of ADH1 and ALDH1, with β-actin expression as an internal control. Densitometric values are shown in the bar graphs. **(E)** Hepatic acetaldehyde concentration. The values shown are average ± SD. *p<0.05, **p<0.005.

The mRNA expression levels of ethanol-metabolizing enzymes alcohol dehydrogenase-1 (ADH1), acetaldehyde dehydrogenase-1 (ALDH1) and cytochrome P450-2E1 (CYP2E1) were found to be similar in pair-fed WT and ALR-deficient mice (Fig 5A–5C). The mRNA expression of all 3 enzymes increased in ethanol-fed WT mice (the magnitude of increase being much greater for ADH1 and ALDH1), but decreased in ALR-deficient mice. Consistent with the mRNA expression, ADH1 protein increased in ethanol-fed WT mice but not in ALR-deficient mice (Fig 5D). ALDH1 protein levels were lower in pair-fed ALR-deficient mice than in WT mice, and ethanol ingestion did not significantly alter ALDH1 protein expression both in WT and ALR-deficient mice (Fig 5D). Hepatic acetaldehyde concentration increased modestly in ethanol-fed WT mice (Fig 5E). Interestingly, acetaldehyde concentration was already greater in pair-fed ALR-deficient mice than in WT mice, and this increased further by alcohol ingestion for 4 weeks (Fig 5E).

### Ethanol augments oxidative stress and mitochondrial dysfunction in ALR-deficient mice

**Fig 6:**
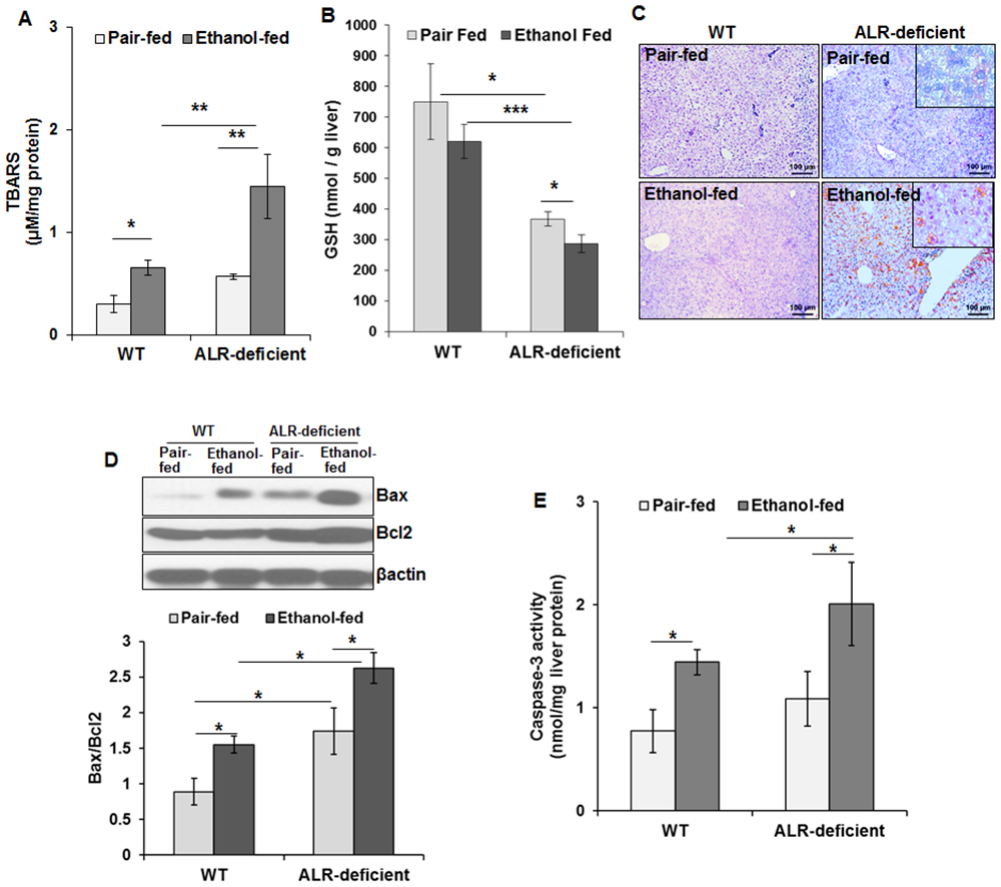
Ethanol exacerbates hepatic oxidative stress, DNA damage, and apoptosis in ALR-deficient mice. WT and ALR-deficient mice were placed on isocaloric- or ethanol-diet for 4 weeks. Bar graphs show changes in hepatic **(A)** TBARS and **(B)** GSH. **(C)** Representative images of the liver sections show 8-oxoguanine staining as a marker for DNA damage (Original magnification, x10). Hepatic expression of Bax and Bcl2 **(D)** and caspase-3 activity **(E)** in ethanol-fed WT and ALR-deficient mice. The values in bar graphs are average ± SD. *p<0.05, **p<0.005, ***p<0.0005.

Ethanol ingestion increased oxidative stress (increased TBARS and decreased GSH) in WT mice (Fig 6A and 6B). Previously, we observed continued low level oxidative stress, inflammation, cell death and regeneration in ALR-deficient mice [15]. Thus, oxidative stress was already apparent in the livers of ALR-deficient mice that was augmented upon ethanol ingestion (Fig 6A and 6B). Mitochondria are a primary site of the generation of reactive oxygen species (ROS), and excessive oxidative stress causes mitochondrial DNA damage and injury. To determine mitochondrial DNA damage due to ethanol, we stained the liver sections for 8-oxoguanine. 8-oxoguanine staining increased strongly in ethanol-fed as compared to the pair-fed ALR-deficient mice (Fig 6C). In contrast, pair-fed or ethanol-fed WT mice did not show any obvious 8-oxoguanine staining.

The pro-apoptotic Bax protein expression increased in ethanol-fed WT as well as ALR-deficient mice as compared to respective pair-fed mice; anti-apoptotic Bcl2 expression did not change in either phenotype upon ethanol feeding causing an increased Bax/Bcl2 ratio (Fig 6D). Bax/Bcl2 ratio was already greater in pair-fed ALR-deficient mice than in WT mice. Although caspase-3 activity was similar in pair-fed WT and ALR-deficient mice, ethanol consumption increased it in both phenotypes, the increase being of a greater magnitude in ALR-deficient mice (Fig 6D).

### Ethanol-fed ALR-deficient mice develop increased inflammation and excessive fibrosis

**Fig 7:**
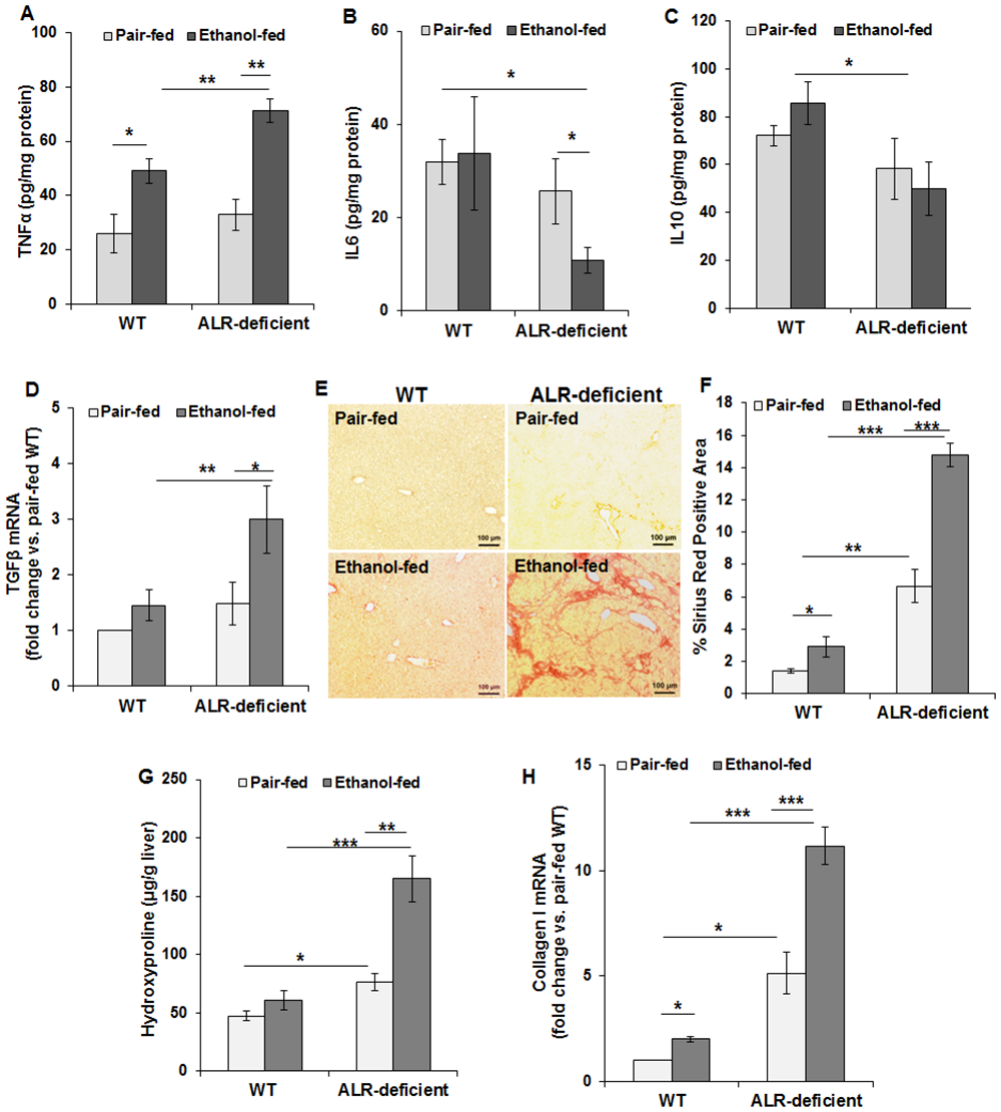
Ethanol exacerbates hepatic inflammation, and strongly augments fibrosis in ALR-deficient mice. WT and ALR-deficient mice were placed on isocaloric- or ethanol-diet for 4 weeks. Bar graphs show changes in hepatic mRNA expression of **(A)** TNFα, **(B)** IL6, **(C)** IL10 and **(E)** TGFβ. **(E)** Representative liver sections showing sirius red staining as an indicator of fibrosis (Original magnification, x10) and **(F)** quantification of sirius red staining. **(G)** Hydroxyproline levels and **(H)** mRNA expression of collagen I in pair-fed and ethanol-fed WT and ALR-deficient mice. The mRNA expression of the respective molecules were normalized with that of β-actin. The values shown are averages ± SD; *p<0.05, **p<0.005, ***p<0.0005.

The increased oxidative stress in ethanol-fed ALR-deficient mice was associated with increased levels of pro-inflammatory cytokine TNFα, and reduced levels of hepatoprotective cytokine IL6 and anti-inflammatory IL10 (Fig 7A–7C). Furthermore, hepatic expression of the potent fibrogenic cytokine TGFβ increased significantly in ethanol-fed ALR-deficient but not in WT mice (Fig 7D). Consistent with this finding, the modest fibrosis seen in pair-fed ALR-deficient mice increased robustly upon ethanol ingestion as determined by sirius red staining (Fig 7E and 7F), hydroxyproline content (Fig 7G) and collagen I mRNA expression (Fig 7H). Pathology scores were also congruent with the histological and biochemical assessments [increase in fibrosis from stage 1 in pair-fed to stage 3 in ethanol-fed ALR-deficient mice (Table 2)].

**Fig 8:**
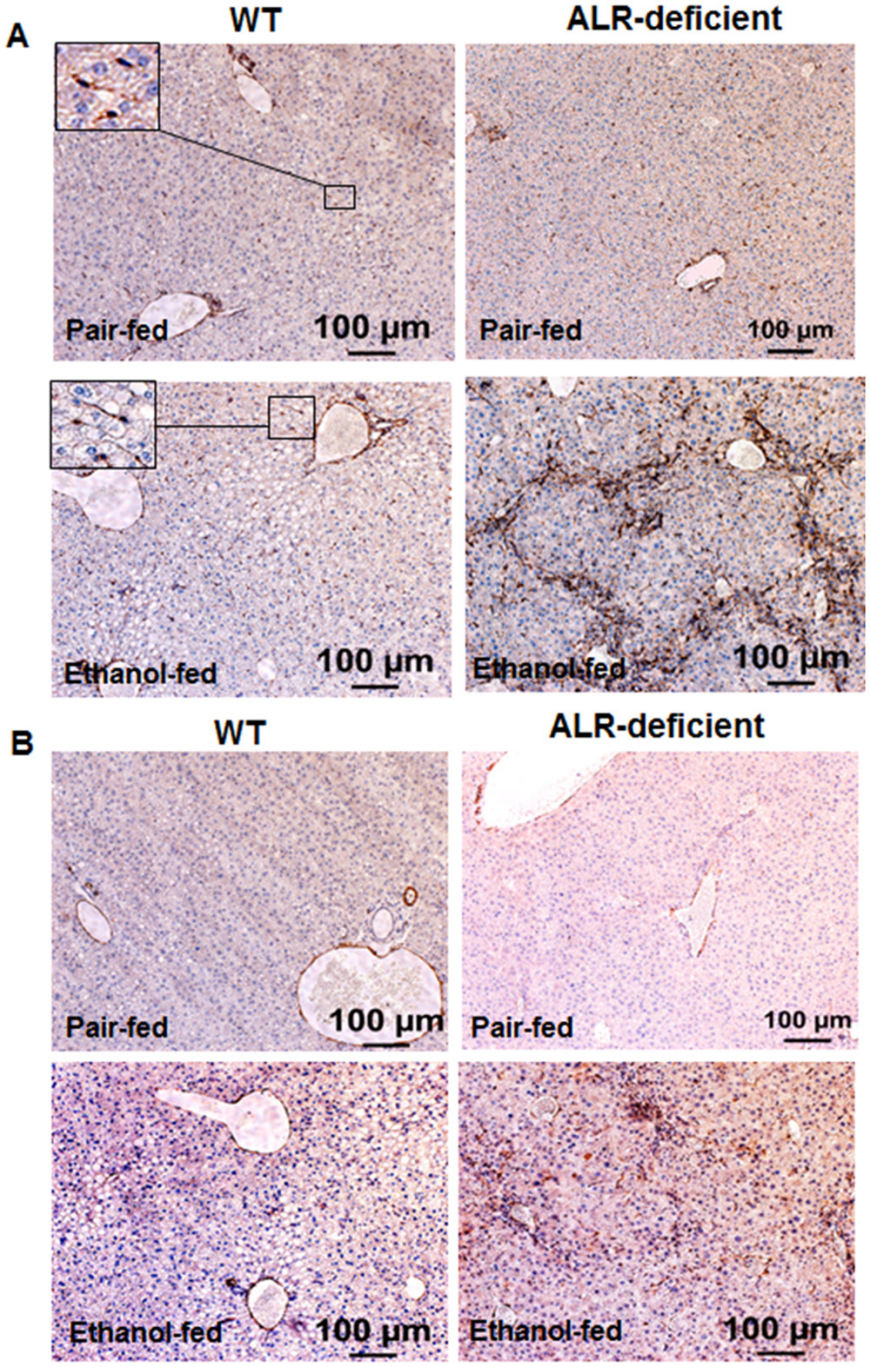
Hepatic desmin and α-SMA expression in alcohol-fed mice. Liver sections of pair-fed and alcohol-fed WT and ALR-deficient mice were immunostained for **(A)** desmin (a marker for HSCs) and **(B)** α-SMA (a marker for activated HSCs) as described in the Materials and Methods section.

While activation of portal fibroblasts to fibrogenic myofibroblasts is an important component of biliary fibrosis [22], overwhelming evidence indicates that the perisinusoidal hepatic stellate cells (HSCs) are the major source of liver fibrosis of any etiology [22, 23]. During liver injury, HSCs transdifferentiate from their quiescent physiologic to the fibrogenic phenotype. Such transformation is induced by inflammatory mediators (e.g., TNFα) and reactive oxygen species and apoptotic bodies arising from dying hepatocytes and activated HSCs are characterized by expression of αSMA, a marker not found in quiescent HSCs in physiology [23, 24]. Interestingly, whereas immunostaining of the liver sections showed large accumulation of desmin-positive cells (an established marker of HSCs) in the fibrous areas of ethanol-fed ALR-deficient mice indicating proliferating HSCs (Fig 8A), relatively fewer cells were found to express αSMA (Fig 8B). Increase in the number of desmin-positive cells was also observed in the portal areas (Fig 8B) suggesting that the biliary component and portal myofibroblasts could be important contributors to enhanced fibrogenesis in alcohol-fed ALR-deficient mice. Only minimal pericellular fibrosis was observed in ethanol-fed WT mice (Table 2).

### Ethanol enhances iron overloading in liver of ALR-deficient mice

**Fig 9:**
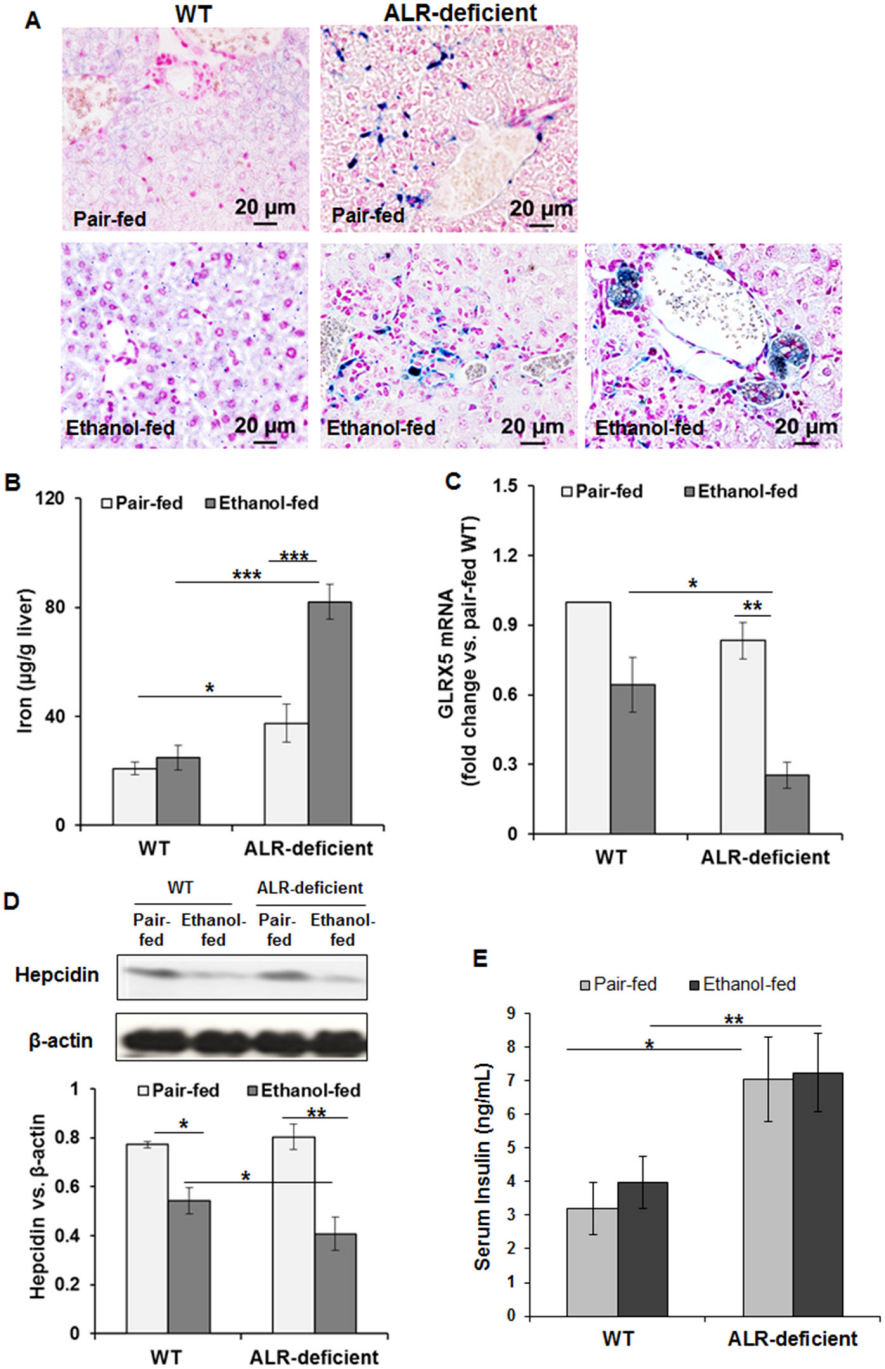
ALR deficiency is associated with iron overloading in ethanol-fed ALR-deficient mice. **(A)** Representative Prussian blue stained images showing iron accumulation in the liver. Original magnification, x40. Bar graphs show hepatic **(B)** iron content and **(C)** glutaredoxin-5 (GLRX5) mRNA expression relative to that of β-actin. **(D)** Western blot showing hepcidin protein expression and its densitometric quantification (bar graph). **(E)** Serum insulin levels. The values are average ± SD. *p<0.05, **p<0.005, and ***p<0.0005.

Because ALR has been implicated in iron homeostasis [11] and alcohol and iron are synergistically hepatotoxic [25–28], we examined iron accumulation in ALR-deficient mice. There was no significant change in iron accumulation in ethanol-fed compared to the pair-fed WT mice (Fig 9A and 9B). In the pair-fed ALR-deficient mice, iron deposits were localized mostly in sinusoidal cells. Ethanol consumption markedly increased iron deposits in periportal and midzonal cells and pigmented macrophages in ALR-deficient mice (Fig 9A). Biochemical analysis confirmed significantly greater hepatic iron content in ethanol-fed as compared to pair-fed ALR-deficient mice (Fig 9B). Analysis of the expression of glutaredoxin-5 (GLRX5), a mitochondrial protein that plays an important role in iron homeostasis, revealed its down-regulation in both WT and ALR-deficient mice, the magnitude being much greater in the latter (Fig 9C). Another important protein that plays a critical role in hepatic iron homeostasis is hepcidin, which is a negative regulator of iron absorption and its release from the reticuloendothelial cells [29]. Analysis of hepcidin showed its reduced expression in both ethanol-fed WT and ALR-deficient mice, the reduction being of greater magnitude in ALR-deficient mice (Fig 9D). Next we measured circulating insulin in pair-fed and alcohol-fed WT and ALR-deficient mice because insulin has been shown to regulate hepatic hepcidin levels and iron overload [30–34]. Insulin levels were significantly higher in pair-fed ALR-deficient mice compared to the WT mice, and they did not change following alcohol feeding in both groups (Fig 9E).

### Lower hepatic ALR levels are associated with advanced ALD in humans

**Fig 10:**
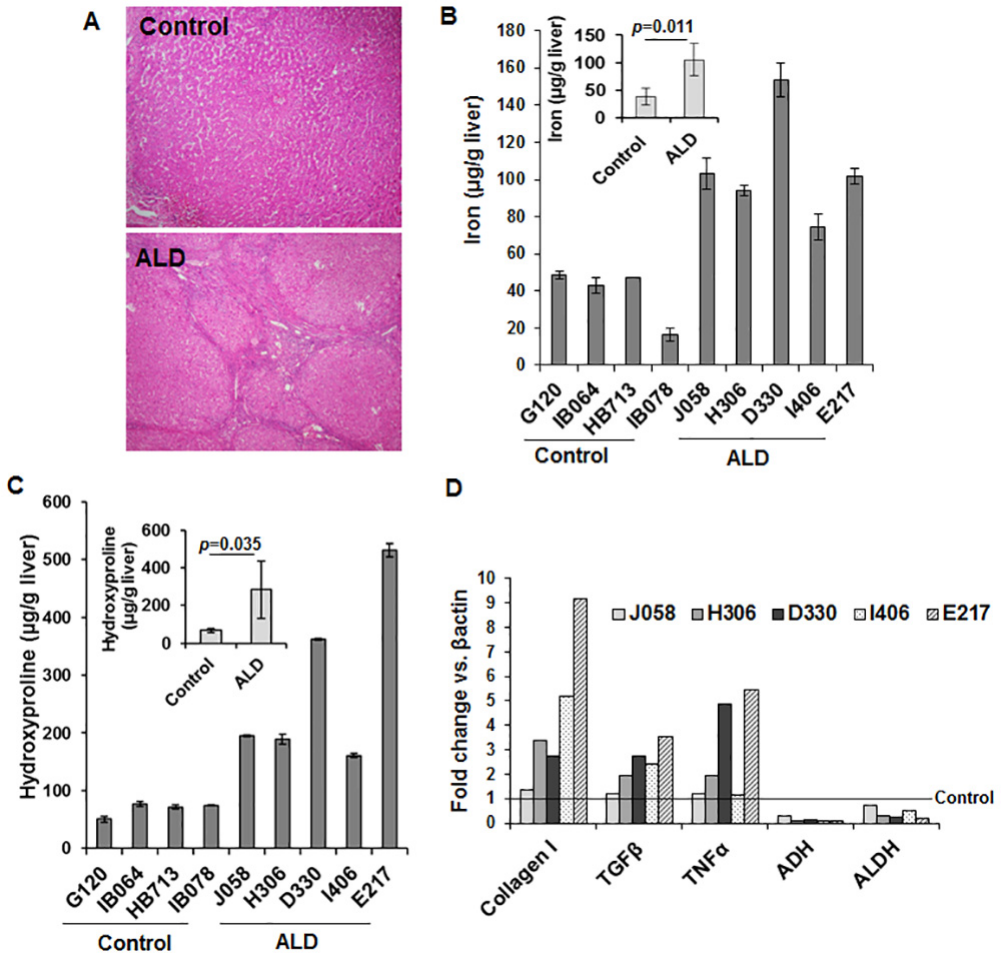
Hepatic iron, fibrogenic markers and alcohol-metabolizing enzymes in human advanced ALD. **(A)** Representative H/E section of the human de-identified advanced ALD liver. Hepatic **(B)** iron and **(C)** hydroxyproline contents, and **(D)** collagen I (COL I), TGFβ, TNFα, ADH and ALDH1 mRNA expression. The mRNA expression of the respective molecules was normalized with that of β-actin.

We previously reported that hepatic ALR was lower in cirrhotic ALD subjects than in control [15]. We assessed relevance of these and findings in ethanol-fed ALR-deficient mice to human advanced ALD (de-identified samples from the Health Sciences Tissue Bank, University of Pittsburgh Medical Center) (Fig 10A). Hepatic iron (Fig 10B) and hydroxyproline content (Fig 10C), and collagen type I and TGF-β1 expression (Fig 10D) were significantly higher, and expression of ADH1 and ALDH (Fig 10D) was lower in ALD compared to the control.

## Discussion

Alcohol-induced liver disease progresses through the development of steatosis, hepatocellular injury and inflammation to fibrosis. However, excessive fibrosis and irreversible cirrhosis occur in only a subpopulation of alcoholics, cause(s) and mechanisms of which have been elusive. Very high frequency of cirrhosis observed at autopsy in alcoholics [35–37] suggests that the underlying liver injury may remain undiagnosed in alcohol abusers with silent progression to irreversible liver damage. In this investigation, we found that liver-specific ALR-deficient mice develop excessive fibrosis upon alcohol ingestion for 4 weeks; WT controls developed only steatosis.

Enhanced lipogenesis and reduced fatty acid oxidation are critical to the development of alcohol-induced steatosis. Increased hepatic accumulation of free fatty acids originating from adipose tissue along with increased SREBP1c and FAS activity has been shown in rodents upon chronic ethanol intake [38]. We found increased hepatic expression of ACACA, SREBP1c and FAS in association with reduced expression/activity of CPT1α in ethanol-fed WT mice. These data suggest that increased de novo synthesis together with inhibition of the transport of fatty acids into mitochondria contribute to steatosis in the WT mice. In contrast, CPT1α expression/activity in the ALR-deficient liver was at the WT basal level and did not change upon ethanol feeding, and expressions of ACACA, SREBP1c and FAS decreased after ethanol. In ALR-deficient mice, these effects of alcohol on enzymes responsible for lipid accumulation are surprising, but it should be noted that steatosis is absent or very low in human alcohol-induced cirrhotic liver (e.g., see Fig 10A). We also note that the severely ALR-deficient mice were found to have strongly reduced expression/activity of CPT1α and robust steatosis at 2 weeks of age, and upon appearance of ALR, CPT1α normalized with resolution of steatosis by 8 weeks [15]. It is plausible that the early occurrence and subsequent normalization of steatosis develops a compensatory mechanism that resists steatosis development during ethanol ingestion in the ALR-deficient mice. How ALR regulates the expression of the enzymes of lipid homeostasis in physiology and during pathological development is a topic of future investigation.

ADH1 has very high affinity for alcohol (K_m_ of 0.1–1 mM); it metabolizes ethanol to acetaldehyde, which is converted to acetate by ALDH. Ethanol ingestion increased ADH1 protein content in WT but not in ALR-deficient mice, and interestingly ALDH1 protein content, that was lower in pair-fed ALR-deficient mice than in WT mice, remained unaltered after alcohol ingestion. These data suggest that hepatic metabolism of ethanol to acetaldehyde and then to acetate occurs efficiently in WT mice, but the much lower ALDH1 in ALR-deficient mice might have caused accumulation of acetaldehyde. Accumulation of acetaldehyde in pair-fed ALR-deficient mice may be due to its defective clearance and/or increased generation from other physiological sources such as pyruvate and threonine. Since acetaldehyde (a) forms stable and unstable forms of adducts with proteins, lipids and DNA [39, 40], (b) promotes oxidative stress by reacting with and by depleting cellular GSH [41], and (c) is also a potent stimulant of collagen synthesis [42, 43], low ALDH in ALR-deficient mice can be an important mechanism of increased inflammation and oxidative stress, and excessive fibrosis. Furthermore, adduct formation of collagen with acetaldehyde may have led to impaired collagen degradation and promoted fibrosis. Since ALDH is decreased in subjects with alcohol-unrelated pre-existing liver disease [44], it may be postulated that ALR deficiency-induced underlying liver injury and low ALDH can be critical mechanisms of the predisposition of these subjects to advanced ALD. Indeed, correlation between decreased ALDH activity and the severity of alcohol-induced liver injury and hepatic fibrosis has been reported [45, 46].

Despite possessing lower catalytic efficiency than ADH, CYP2E1 can be an important enzyme of ethanol metabolism in the liver during chronic or excessive alcohol consumption [47–50]. Consistent with these findings, we observed increased CYP2E1 expression in ethanol-fed WT mice. Increased CYP2E1 is cytotoxic to hepatocytes during chronic ethanol feeding because it promotes oxidative stress [51, 52], and initiates peroxidation of membrane lipids in the presence of small amounts of non-heme iron [53]. Although significantly reduced expression of CYP2E1 in ethanol-fed ALR-deficient mice indicates that this enzyme may not be of significance in ethanol-induced pathological development in them, a recent study conducted in a Chinese population found that subjects with alcoholic cirrhosis have lower CYP2E1 expression than healthy individuals [54].

Mitochondria are important source of ROS generation [55]. Ethanol-induced production of ROS (H_2_O_2_, OH^−^ and O_2_^−^ radicals) [56] alters cytosolic and mitochondrial redox state [57]. Much greater levels of TBARS and lower GSH/GSSG ratio in ethanol-fed ALR-deficient mice are indicative of excessive hepatic oxidative stress, which can be damaging to cellular and mitochondrial DNA. Indeed, cytosolic and mitochondrial 8-oxoguanine binding increased in ethanol-fed ALR-deficient mice. These results are consistent with our previous findings showing maintenance of redox balance in mitochondria of isolated hepatocytes by ALR, and apoptosis of hepatocytes upon ALR depletion [15]. Congruent with these observations, oxidative stress and mitochondrial damage reflected into increased activation of pro-apoptotic signaling likely resulting in hepatocyte apoptosis in alcohol-fed ALR-deficient mice.

The two cell types that contribute to liver fibrosis are HSCs and portal fibroblasts, and activation of these cells to myofibroblasts is critical to their fibrogenic function [22, 23]. The role of epithelial-to-mesenchymal transition in hepatic fibrosis has been shown to be minimal at best [22, 58–60]. Recent work involving fate-tracing studies suggest that as the disease progresses, activated HSCs become major cell type of liver fibrosis in both bile duct ligation- and toxin-induced liver fibrosis [59]. Whether such may be the case for alcohol-induced fibrosis is not known. However, strong correlation of alcohol-induced fibrosis in human liver disease with ductular proliferation and portal inflammation [21], and our observation showing heightened fibrosis in association with increased portal inflammation and ductular proliferation, and increased number of desmin-positive cells in the portal regions, (Fig 3B; S2 Fig; Table 2) of the alcohol-fed ALR-deficient mice suggest that portal fibroblasts can continue to be important in alcohol-induced liver fibrosis.

Liver plays a critical role in iron homeostasis, and although it has large iron-storing capacity, excessive iron deposition is a major mechanism of hepatic injury including steatohepatitis, fibrosis/cirrhosis and liver cancer. Iron homeostasis is essential for maintenance of functional integrity of mitochondria; its dysregulation increases cellular iron uptake leading to mitochondrial damage [61]. Here, we observed pre-existing hepatic iron deposits in ALR-deficient mice, which increased further upon ethanol feeding. These data suggest that ALR may play an essential role in regulating iron homeostasis, and its loss can be an important mechanism of iron overload causing oxidative damage via iron-catalyzed oxidation reactions [62, 63]. In this regard, we found significant down-regulation of GLRX5 and hepcidin in ethanol-fed as compared to pair-fed ALR-deficient mice. GLRX5 is a Fe–S cluster assembly gene, loss of which disrupts the regulation of iron homeostasis [64]. Splicing defect in GLRX5 has been shown to cause severe anemia, enlargement of liver and spleen, and hepatic iron overload associated with cirrhosis [65]. Hepcidin, a peptide hormone primarily produced by hepatocytes [66], plays a key role in the regulation of systemic iron homeostasis, and its down-regulation has been postulated as a possible mechanism leading to iron overloading in ALD [67, 68]. Decreased hepcidin mRNA expression in mice has been shown to result in iron overloading, similar to human hemochromatosis [69]. We observed minimal iron deposition in WT mice after ethanol feeding despite reduced hepcidin, but iron deposition was excessive in ALR-deficient mice. Thus, it is apparent that ALR could play an important role in iron homeostasis via GLRX5 and/or hepcidin regulation. A caveat of these findings is that despite down-regulation of glutaredoxin-5 and hepcidin in WT ethanol-fed mice, there was no significant increase in iron deposition. It is likely that up to a threshold level, the decrease in GLRX5 and hepcidin maintain iron homeostasis, or other parameters associated with underlying hepatic injury in ALR-deficient mice could be contributing factors for aberrant iron deposition upon ethanol ingestion.

Evidence has accumulated indicating a role for insulin in regulation of hepcidin expression and iron overload. Insulin resistance occurring in metabolic syndrome and insulin deficiency in diabetes have been shown to be associated with hepatic iron overload [30–33]. However, despite unaltered insulin levels hepcidin expression decreased in ethanol-fed WT mice, and a further reduction in its expression in ethanol-fed ALR-deficient mice occurred without any change in already increased insulin levels. We note that hepatic iron accumulation occurred in high fat/high fructose-fed mice prior to insulin resistance and down-regulation of hepcidin [32]. It seems that the relationship between insulin, hepcidin and iron homeostasis is quite complex with mechanisms specific for the underlying pathological progression. Thus, a detailed analysis of the association of ALR deficiency, changes in insulin levels, iron overload and fibrosis/cirrhosis with GLRX5 and hepcidin reduction in the presence of underlying liver injury warrants future investigation.

In summary, the results of the present investigation demonstrate that ongoing inflammation and liver injury due to ALR deficiency is augmented strongly upon ethanol ingestion. The defective ethanol metabolism due to low ALDH activity may be a major factor driving pathological changes leading to excessive fibrosis. Although Lower ALR, ADH and ALDH expression in human liver samples from patients with end-stage alcohol-induced liver disease indicate a correlation between ALR and ethanol-metabolizing enzymes, whether lower ALR is a consequence of liver disease or ALR anomaly/deficiency drives progression to the fibrogenic liver disease remains to be determined. Nevertheless, it can be postulated that ALR deficiency or anomaly may be an important determinant of the predisposition of humans to alcoholic cirrhosis.

## Supporting Information

S1 Fig Western blot showing hepatic ALR in nonreducing and reducing conditions. Liver protein lysates were prepared in RIPA buffer (Santa Cruz Biotechnology) and proteins were separated on a 15% SDS-PAGE under nonreducing or reducing conditions. The separated proteins were transferred on to Immobilon^®^–P PVDF membrane. Following treatment with polyclonal rabbit anti-GFER Ab (Proteintech Group, Chicago, IL), detection was achieved using ECL reagent (Thermo Fisher, Grand Island, NY).
(TIF)

S2 Fig Enhanced ductular proliferation in ethanol-fed ALR-deficient mice. WT and ALR-deficient mice were fed isocaloric or alcohol diet for 4 weeks. Paraffin-embedded liver sections were deparaffinized followed by microwave heating in citrate buffer (pH 6.0). The sections were then incubated with goat polyclonal keratin 19 Ab (sc33119, Santa Cruz Biochemicals, CA, USA) at 1:50 dilution overnight at 4°C, followed by incubation with anti-Goat IgG-HRP (Sigma, USA) for 2h at room temperature, then reacted with diaminobenzidine (DAB, Thermo Scientific) to develop brown color.
(TIF)

S3 Fig Chow diet-fed WT and ALR-deficient mouse liver at 12 weeks. H/E-stained liver sections of chow diet-fed WT and ALR-deficient mice at 13 weeks of age (equivalent of alcohol- or pair-fed groups). Ductular proliferation is minimal or not apparent in ALR-deficient mice.
(TIF)

## Abbreviations

ACACA: acetyl-CoA carboxylase
ADH: alcohol dehydrogenase
ALD: alcoholic liver disease
ALDH: acetaldehyde dehydrogenase
ALR: augmenter of liver regeneration
CPT1α: carnitine palmitoyl transferase-1α
CYP2E1: cytochrome P450-2E1
FAS: fatty acid synthase
GLRX: glutaredoxin
IL: interleukin
ROS: reactive oxygen species
SREBP: sterol regulatory element-binding protein
TBARS: thiobarbituric acid reactive substances
TGF: transforming growth factor
TNF: tumor necrosis factor
WT: wild type
