# Supplementary figures and images for: Hepatic Deficiency of Augmenter of Liver Regeneration Exacerbates Alcohol-Induced Liver Injury and Promotes Fibrosis in Mice
Source: PLoS One. 2016 Jan 25;11(1):e0147864. doi: 10.1371/journal.pone.0147864 (PMC4726524; doi:10.1371/journal.pone.0147864)

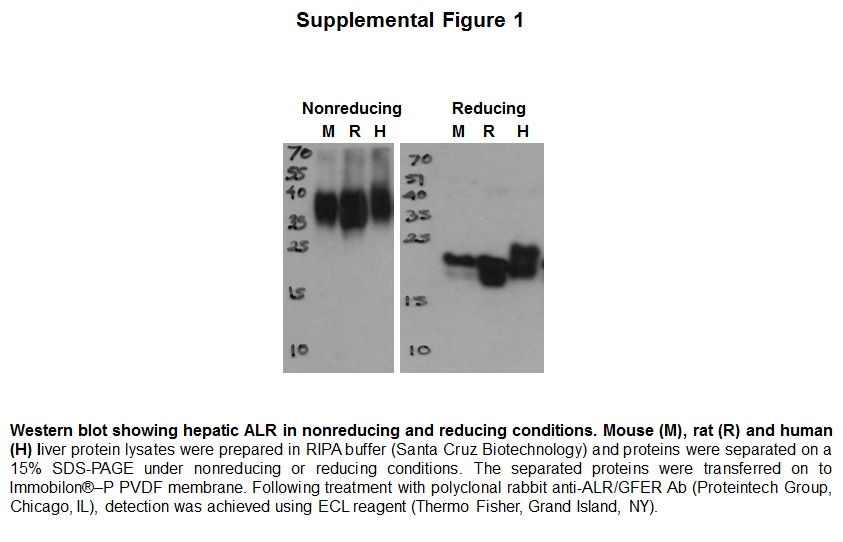

Supplement: S1 Fig — Liver protein lysates were prepared in RIPA buffer (Santa Cruz Biotechnology) and proteins were separated on a 15% SDS-PAGE under nonreducing or reducing conditions. The separated proteins were transferred on to Immobilon®–P PVDF membrane. Following treatment with polyclonal rabbit anti-GFER Ab (Proteintech Group, Chicago, IL), detection was achieved using ECL reagent (Thermo Fisher, Grand Island, NY). (TIF) [file pone.0147864.s001.tif]

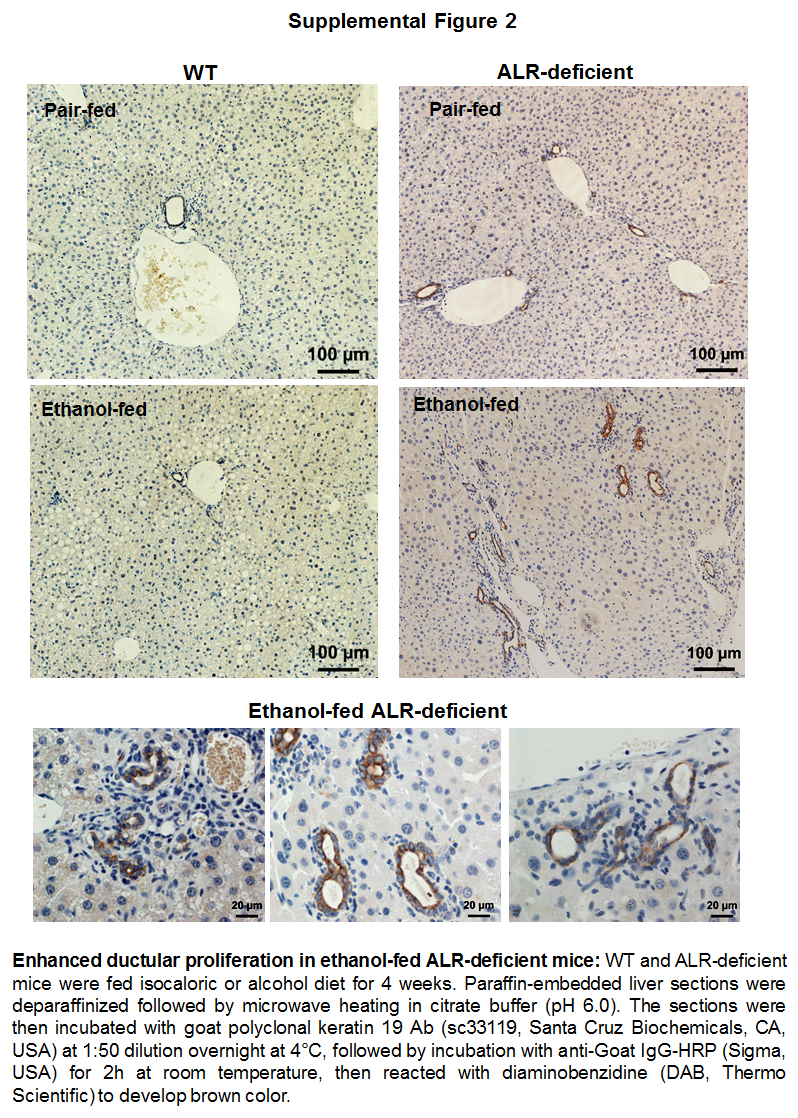

Supplement: S2 Fig — WT and ALR-deficient mice were fed isocaloric or alcohol diet for 4 weeks. Paraffin-embedded liver sections were deparaffinized followed by microwave heating in citrate buffer (pH 6.0). The sections were then incubated with goat polyclonal keratin 19 Ab (sc33119, Santa Cruz Biochemicals, CA, USA) at 1:50 dilution overnight at 4°C, followed by incubation with anti-Goat IgG-HRP (Sigma, USA) for 2h at room temperature, then reacted with diaminobenzidine (DAB, Thermo Scientific) to develop brown color. (TIF) [file pone.0147864.s002.tif]

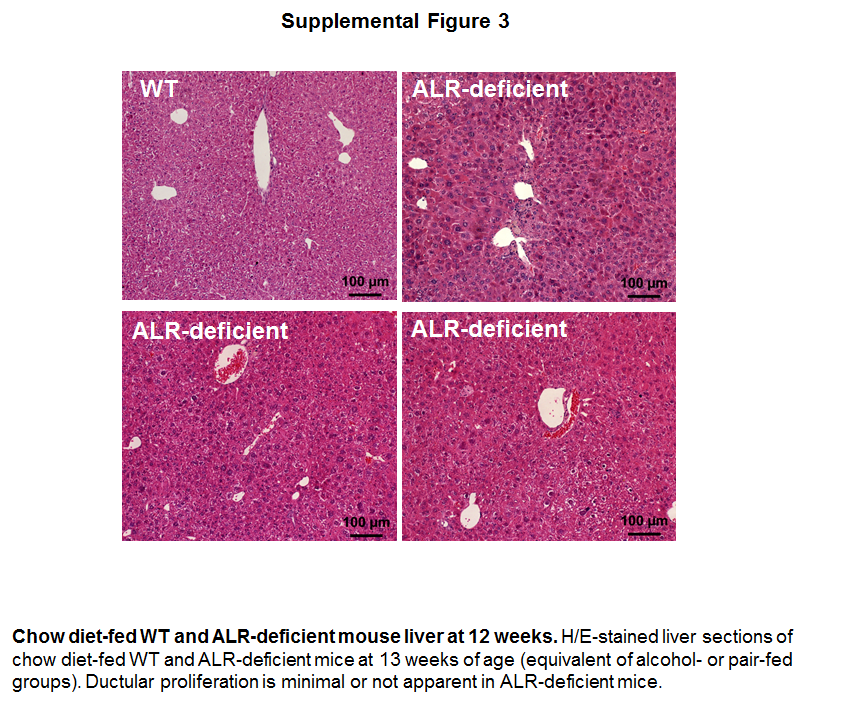

Supplement: S3 Fig — H/E-stained liver sections of chow diet-fed WT and ALR-deficient mice at 13 weeks of age (equivalent of alcohol- or pair-fed groups). Ductular proliferation is minimal or not apparent in ALR-deficient mice. (TIF) [file pone.0147864.s003.tif]
